# Brazilian Pediatric Reference Data for Quantitative Ultrasound of Phalanges According to Gender, Age, Height and Weight

**DOI:** 10.1371/journal.pone.0127294

**Published:** 2015-06-04

**Authors:** Ezequiel Moreira Gonçalves, Roberto Regis Ribeiro, Wellington Roberto Gomes de Carvalho, Anderson Marques de Moraes, Everton Paulo Roman, Keila Donassolo Santos, Pedro Augusto Rodrigues Medaets, Nélio Neves Veiga-Junior, Adrielle Caroline Lace de Moraes Coelho, Tathyane Krahenbühl, Leticia Esposito Sewaybricker, Antonio de Azevedo Barros-Filho, Andre Moreno Morcillo, Gil Guerra-Júnior

**Affiliations:** 1 Growth and Development Lab—Center for Investigation in Pediatrics (CIPED)—School of Medical Sciences (FCM)—University of Campinas (UNICAMP), Campinas-SP, Brazil; 2 Department of Physical Education—Assis Gurgacz Faculty, Cascavel-PR, Brazil; 3 Department of Physical Education—Center for Biological Sciences and Health—Federal University of Maranhão (UFMA), São Luís-MA, Brazil; 4 Department of Pediatrics—FCM—UNICAMP, Campinas-SP, Brazil; Garvan Institute of Medical Research, AUSTRALIA

## Abstract

**Aims:**

To establish normative data for phalangeal quantitative ultrasound (QUS) measures in Brazilian students.

**Methods:**

The sample was composed of 6870 students (3688 females and 3182 males), aged 6 to 17 years. The bone status parameter, Amplitude Dependent Speed of Sound (AD-SoS) was assessed by QUS of the phalanges using DBM Sonic BP (IGEA, Carpi, Italy) equipment. Skin color was obtained by self-evaluation. The LMS method was used to derive smoothed percentiles reference charts for AD-SoS according to sex, age, height and weight and to generate the L, M, and S parameters.

**Results:**

Girls showed higher AD-SoS values than boys in the age groups 7–16 (p<0.001). There were no differences on AD-SoS Z-scores according to skin color. In both sexes, the obese group showed lower values of AD-SoS Z-scores compared with subjects classified as thin or normal weight. Age (r^2^ = 0.48) and height (r^2^ = 0.35) were independent predictors of AD-SoS in females and males, respectively.

**Conclusion:**

AD-SoS values in Brazilian children and adolescents were influenced by sex, age and weight status, but not by skin color. Our normative data could be used for monitoring AD-SoS in children or adolescents aged 6–17 years.

## Introduction

The adequate acquisition of bone mass during childhood and adolescence leads to a healthy bone structure throughout life [1, 2]. Therefore, bone mass assessment at those ages represents a useful tool to identify individuals who may have a greater risk of osteoporosis in adult life [3–4].

Quantitative ultrasound (QUS) of the proximal phalanges has been used for indirect assessment of bone tissue. Several experiments suggest that ultrasound parameters provide information not only on quantity, but also on the architecture and elasticity of bone [5–9]. Furthermore, this method has practical advantages compared to conventional methods which uses x-rays and photons, i.e. dual-energy X-ray absorptiometry (DXA), and peripheral quantitative computed tomography. It is safe, relatively cheap, takes less time to measure, the equipment is portable and ionizing radiation-free, which makes a good indication for its use with children and adolescents [5, 10–12].

Percentile reference charts are widely used in medical practice as a screening tool to identify how a value of an individual or a sample is positioned compared to the normal distribution of a reference population [13]. Most studies with children and adolescents to provide normative data for the QUS parameters, such as Amplitude Dependent Speed of Sound (AD-SoS), are based on European samples [14–20]. Those references may lead to contradictory results when used in populations belonging to other countries such as Brazil [21–23]. Reference growth charts are needed when any measurement depends strongly on co-variables such as sex, age, pubertal development, as it is observed for QUS parameters of phalanges [11,15,18,20,24].

Several factors influences bone mass mineralization such as sex, ethnicity, heredity, hormonal processes, diet, physical activity level and body weight. The latter has been an important concern and recent studies observed that, regardless of the method used to assess bone status (QUS or DXA), there is a negative influence of obesity on bone health in pediatric ages [25,26]. Thus, the aims of the present study were to establish AD-SoS smoothed percentiles assessed by QUS of the phalanges in Brazilians students aged 6 to 17 years according to sex, age, height and weight, and to verify the influence skin color, and weight status on bone status of these children and adolescents.

## Materials and Methods

Measurements were performed on 6,870 children and adolescents (3,688 girls and 3,182 boys), aged 6 to 17 years, recruited in schools from three cities located in the state of Paraná (*Cascavel*: n = 3,504; 2,016 females and 1,488 males; *Ceu Azul*: n = 757; 388 females and 369 males; and *Vera Cruz*: n = 784; 401 females and 383 males), and from two cities in the state of Sao Paulo (*Campinas*: n = 1,296; 633 females and 663 males; and *Francisco Morato*: n = 529; 250 females and 279 males). Paraná and São Paulo are located in the south and southeast regions of Brazil, respectively. The data were collected between 2006 and 2010 and results of each city have already been published [21,22,27,28]. Exclusion criteria were the presence of physical deficiencies (permanent or temporary) which made assessment impossible, use of drugs that could interfere with bone mass, non-agreement of parents or students, not turning up on the assessment day. According to those criteria 122 students were excluded. All subjects and their parents or guardians were informed about the possible risks of the investigation before giving written informed consent to participate. All procedures were approved by the ethics committee of the School of Medical Sciences, University of Campinas, and were conducted in accordance with the declaration of Helsinki for human studies.

Decimal age was calculated as the difference between date of birth and date of data collection. Each age group was categorized by the midpoint of an age range. For example, the group of children with 8 years old included all the children between 7.50 and 8.49 years, and so forth. The same calculation was used to determine the categories of height and weight (e.g. 150 cm = 145 to 154.9 cm and 50 kg = 45 to 54.9 kg).

Self-reported skin color was collected based on the Brazilian Institute of Geography and Statistics (IBGE) categories [29]: white,” “*mulatto*” (lighter skinned black), and black. The results of those who reported as having yellow or indigenous skin color altogether accounted for less than 10% of the sample and therefore are not shown in tables but were included in the association analyses.

All anthropometric measurements were performed according to standardized procedures [30]. Weight was measured (kg) using portable digital scales to the nearest 0.1 kg. Height was measured (cm) using a vertical stadiometer to the nearest 0.1 cm. From those measurements, BMI was calculated using the formula (kg/m^2^). Height and BMI values were converted to standard deviation scores (Z-scores) format by using international reference data [31,32]. We had used the International Obesity Task Force (IOTF) BMI cut-offs to assess the prevalence of thinness, overweight and obesity [32].

For the determination of AD-SoS, DBM Sonic Bone Profiler equipment (IGEA, Carpi, Italy) was used. This equipment is fitted with a probe that attaches two transducers (transmitter and receiver). The probe is positioned at the distal metaphysis of each of the last four proximal phalanges (II to V) in the non-dominant hand, as recommended by the manufacturer.The transducer transmitter emits a sound wave of 1.25 MHz, and the transducer receiver receives the signal and assesses the speed of propagation of sound through the phalange. AD-SoS was obtained automatically and represents the average of speed measurements of ultrasound (m/s) that track the trabecular bone tissue on the four proximal phalanges by transmission. This parameter depends on the amplitude of the electrical signal, obtained after the ultrasound has covered three types of bone in the phalanges (endosteal, trabecular and cortical). All assessments were performed using the same equipment and transducer, calibrated daily according to the manufacturer's recommendations. All the ultrasound measurements were performed by two evaluators (E.M.G. and R.R.R.) using the same equipment. *In vivo* short-term precision was assessed based on root mean square of coefficient of variation (RMS-CV) for 80 measurements made in 10 healthy young persons (6 males and 4 females measured 4 times each by each evaluator) calculated accordingly to Bonnick et al. [33]. The RMS-CV of AD-SoS ranged 0.55 to 0.62% and the inter-evaluator RMS-CV was 1.46%.

To calculated percentiles according to sex and age, the results of the QUS parameters were smoothed using the LMS method [13,34]. L, M and S values were calculated using the software LMSchartmaker Light version 2.42 [35]. Data obtained by the LMS method were converted to Z-scores using the equation: Z = [(X/M)^L^ −1]/(L×S); where X is the measurement value (AD-SoS) and L, M and S are values from the smooth charts for age, height and weight, according to sex (S1, S2 and S3 Tables).

The SPSS version 16.0 (Statistical Package for the Social Sciences, Chicago, IL, USA) was used for database and statistical analysis. The results were expressed as mean, standard deviation (mean ± SD), range (minimum and maximum values) and absolute (n) and relative (%) frequencies. The normal distribution of the data was tested using the Kolmogorov-Smirnov test. We tested several transformations to achieve data normality and homogeneity. However, no approaches produced good outcomes. Therefore, Mann-Whitney U test was used to compare variables between sexes and Kruskal Wallis test was used to compare AD-SoS and Z-scores between age groups, skin color and weight status. Non parametric multiple comparison test and Bonferroni correction were performed when necessary. Pearson´s correlation coefficient was performed to determine correlation between AD-SoS and independent variables. Multiple regression analysis (stepwise method) was performed to identify which variable or combination of variables would best explain AD-SoS variance; for this analysis, data were transformed to reach the parametric tests criteria. The adjusted coefficient of determination (r^2^) was estimated. For all tests, statistical significance was established at p < 0.05.

## Results

General features of the sample are reported in Table 1. Females were significantly older and heavier than males. However, height (cm and Z-scores) and BMI Z-scores were higher in boys. There was no sex related differences for BMI (kg/m^2^) values. The majority of the sample was classified as white skin color (68%), and normal weight status (71%).

**Table 1:** General characteristics of the sample.

	Girls		Boys		All
Variables	Mean ± SD	(range)	Mean ± SD	(range)	Mean ± SD
Age (years) ^a^	11.9 **±** 2.7	(5.5 to 17.5)	11.1 **±** 2.8	(5.5 to 17.5)	11.5 **±** 2.75
Weight (kg) ^a^	41.9 **±** 13.2	(15.0 to 93.7)	41.0 **±** 15.4	(16.0 to 115.5)	41.5 **±** 14.3
Height (cm) ^a^	145.0 **±** 14.3	(105.0 to 183.5)	145.8 **±** 17.3	(105.6 to 194.9)	147.0 **±** 15.8
BMI (kg/m^2^)	18.6 **±** 3.5	(12.0 to 35.5)	18.6 **±** 3.5	(12.5 to 37.7)	18.6 **±** 3.5
Height Z-score (SDS) ^a^	0.23 **±** 1.03	(-3.76 to 5.11)	0.35 **±** 1.09	(-5.57 to 4.82)	0.28 **±** 1.06
BMI Z-score (SDS) ^a^	0.23 **±** 1.03	(-3.02 to 3.25)	0.45 **±** 1.04	(-2.99 to 3.96)	0.33 **±** 1.04
	n (%)		n (%)		n (%)
Skin color/race					
White	2,621 (71.1)		2,064 (64.9)		4,865 (68.2)
Black	324 (8.8)		343 (10.8)		667 (9.7)
Mulatto	702 (19.0)		713 (22.4)		1,415 (20.6)
Yellow/Indigenous	41 (1.1)		62 (1.9)		103 (1.5)
Weight status^b^					
Thinness	422 (11.4)		239 (7.5)		661 (9.6)
Normal	2,646 (71.7)		2,246 (70.6)		4,892 (71.2)
Overweight	492 (13.3)		533 (16.8)		1,025 (14.9)
Obese	128 (3.5)		164 (5.2)		292 (4.3)
Total	3,688 (53.7)		3,182 (46.3)		6,870 (100)

^a^Differences between sexes, p<0.001, Mann-Whitney Test.

^b^Weight status established according to BMI Z-score cut-offs defined by IOTF: Thinness: <-1.01 and <0.98; Normal: between -1.01 to 1.30 and -0.98 to 1.23; Overweigh: between 1.31 to 2.28 and 1.24 to 2.18 and Obese: >2.28 and >2.18 for boys and girls, respectively.

Females showed higher AD-SoS values than males the age groups 7–16 (p<0.001). Significant increase in AD-SoS values was seen when comparing a specific age group with a one-year younger group. This was found for girls from age groups of 10, 12, 13 e 14 years and boys with 9 and 14 years (Table 2).

**Table 2:** AD-SoS values according to sex and age.

			AD-SoS (m/s)			
	Sample size	Girls			Boys	
Age	n (%)	Mean ± SD	(Range)	n (%)	Mean ± SD	(Range)
6	84 (2.3)	1893 ± 66	(1651–2002)	103 (3.2)	1884 ± 64	(1636–1976)
**7** ^**c**^	150 (4.1)	1898 ± 63	(1630–2012)	239 (7.5)	1881 ± 57	(1663–1995)
**8** ^**c**^	167 (4.5)	1919 ± 52	(1714–2040)	331 (10.4)	1880 ± 64	(1638–1991)
**9** ^**c**^	334 (9.1)	1933 ± 53	(1730–2096)	308 (9.7)	**1902 ± 60** ^**a**^	(1650–2069)
**10** ^**c**^	421 (11.4)	**1951 ± 56** ^**a**^	(1767–2119)	388 (12.2)	1911 ± 61	(1644–2039)
**11** ^**c**^	489 (13.3)	1964 ± 50	(1788–2146)	386 (12.1)	1920 ± 64	(1635–2146)
**12** ^**c**^	515 (14.0)	**1985 ± 60** ^**b**^	(1818–2229)	350 (11.0)	1938 ± 57	(1743–2143)
**13** ^**c**^	487 (13.2)	**2017 ± 62** ^**b**^	(1818–2209)	361 (11.3)	1949 ± 71	(1627–2268)
**14** ^**c**^	370 (10.0)	**2046 ± 66** ^**b**^	(1738–2236)	307 (9.6)	**1992 ± 76** ^**b**^	(1767–2319)
**15** ^**c**^	310 (8.4)	2062 ± 57	(1856–2220)	203 (6.4)	2018 ± 78	(1842–2360)
**16** ^**c**^	226 (6.1)	2085 ± 52	(1926–2198)	136 (4.3)	2047 ± 73	(1868–2274)
17	135 (3.7)	2087 ± 53	(1920–2226)	70 (2.2)	2090 ± 64	(1968–2315)
**All** ^**c**^	3688	1992 ± 80	(1630–2236)	3182	1,936 ± 83	(1,627–2,360)

^a^ Significant differences to previous age, p<0.05.

^b^ Significant differences to previous age, p<0.001.

^c^ Significant differences between sexes, p<0.001.

**Fig 1:**
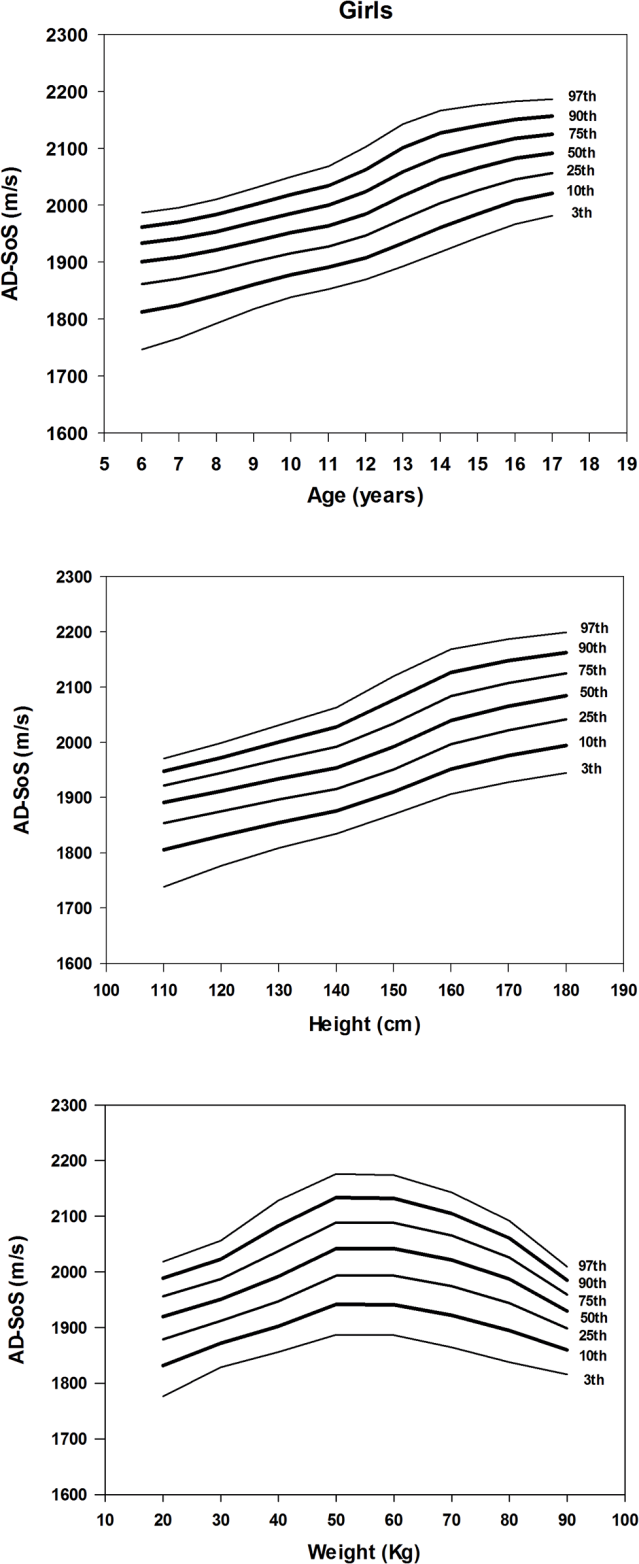
Smoothed percentile charts of AD-SoS for girls according to age (year), height (cm) and weight (kg).

**Fig 2:**
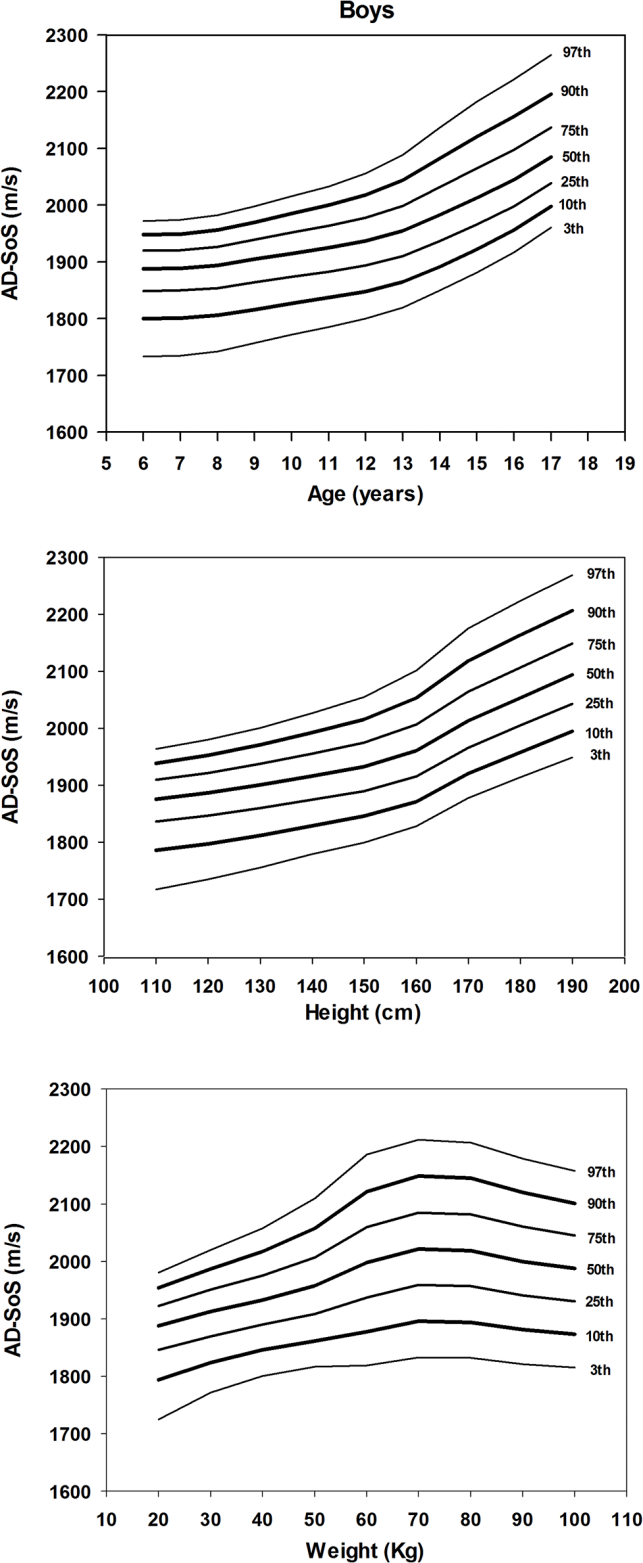
Smoothed percentile charts of AD-SoS for boys according to age (year), height (cm) and weight (kg).

Figs 1 and 2 illustrate the LMS smothed percentiles for AD-SoS according to age, height and weight for girls and boys, respectively. These results (LMS coefficients and percentiles) are presented in detail in the S1, S2 and S3 Tables. In both genders, AD-SoS increased with age and height. However, AD-SoS seems to decrease among girls with body weight above 60 kg and boys with more than 70 kg.

Table 3 shows the AD-SoS Z-scores according to skin color and weight status. There were no differences on AD-SoS Z-scores related to skin color in both sexes. The obese group showed lower values of AD-SoS Z-scores compared with subjects classified as thin and normal weight. Furthermore, overweight girls showed significantly lower AD-SoS Z-scores than thinness and normal weight girls but higher than obese individuals.

**Table 3:** AD-SoS Z-scores according to skin color and weight status.

	AD-SoS Z-score					
		Girls			Boys	
	N	Mean ± SD	Median (range)	N	Mean ± SD	Median (range)
Skin color						
White	2621	-0.01 ± 0.98	-0.03 (-3.29 to 3.55)	2,064	-0.02 ± 0.98	0.01 (-4.84 to 4.60)
Black	324	0.05 ± 1.06	-0.03 (-3.14 to 3.18)	343	0.05 ± 1.09	0.06 (-3.36 to 4.69)
Mulatto	702	0.02 ± 1.07	-0.01 (-4.61 to 4.14)	713	0.03 ± 1.01	0.01 (-3.39 to 3.14)
Yellow/Indigenous	41	-0.18 ± 0.89	-0.06 (-2.66 to 1.43)	62	0.09 ± 1.14	1.00 (-2.62 to 3.54)
Weight status						
Thinness	422	**0.11 ± 1.00** ^**a**^ ^**,**^ ^**b**^	**0.11 (-3.14 to 3.05)**	239	**-0.02 ± 0.93** ^**c**^	**0.00 (-2.92 to 4.04)**
Normal	2,646	**0.07 ± 0.97** ^**a**^ ^**,**^ ^**b**^	**0.03 (-3.29 to 4.14)**	2,246	**0.04 ± 0.99** ^**a**^ ^**,**^ ^**d**^	**0.06 (-3.54 to 4.69)**
Overweight	492	**-0.25 ± 1.01** ^**a**^	**-0.28 (-4.61 to 3.55)**	533	-0.08 ± 1.10	-0.11 (-4.84 to 4.02)
Obese	128	-0.88 ± 1.07	-0.92 (-3.21 to 3.17)	164	-0.28 ± 0.89	-0.29 (-2.68 to 2.49)

^a^ Significant differences from obese, p<0,001

^b^ Significant differences from overweight, p<0,001

^c^ Significant differences from obese, p<0,05

^d^ Significant differences from overweight, p<0,01.

**Fig 3:**
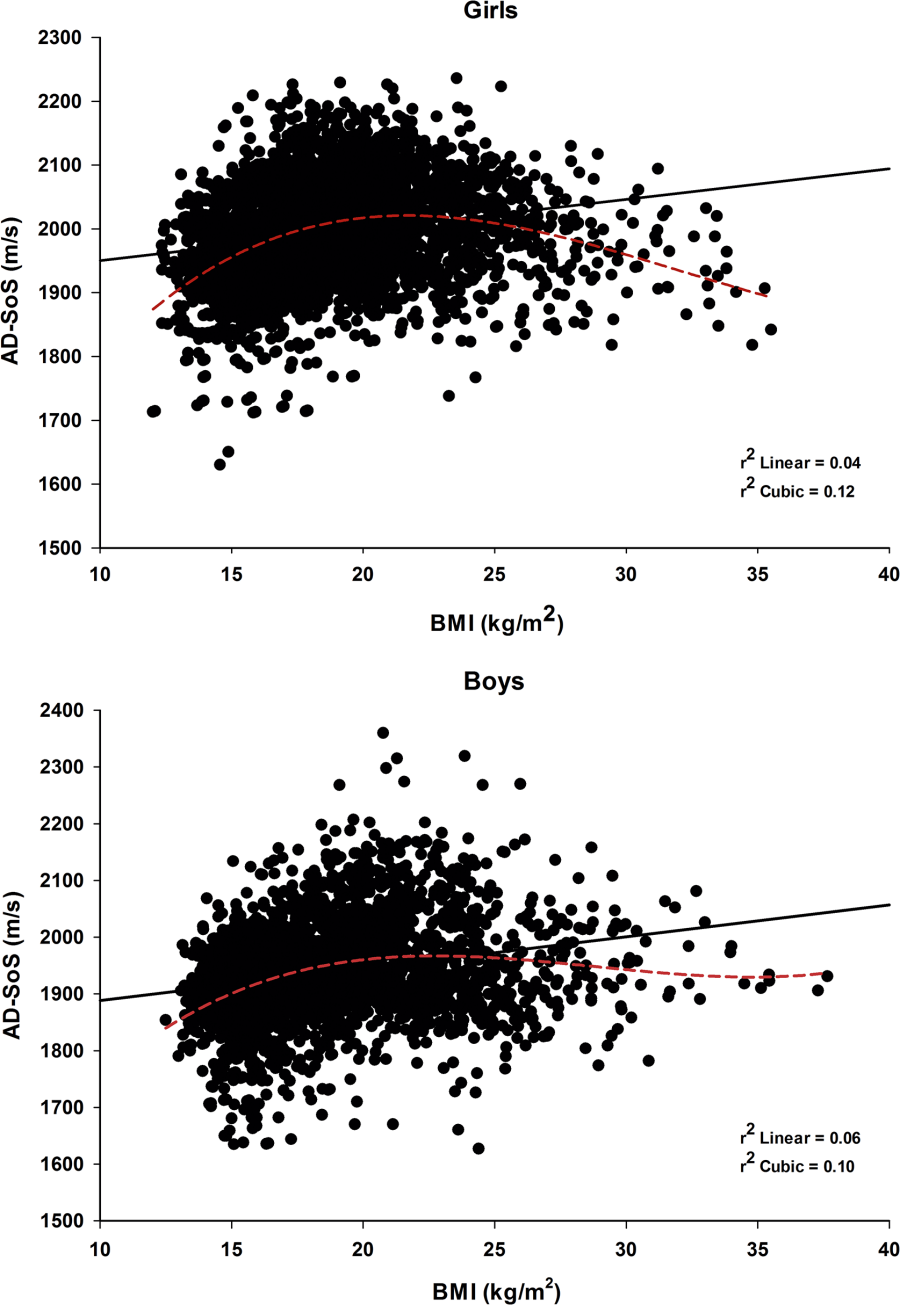
Linear (solid line) and cubic (dotted line) relationship between AD-SoS and BMI for girls and boys.

Fig 3 illustrates the relationship between AD-SoS and BMI for girls and boys.

Positive correlation was observed between AD-SoS and BMI both in girls and boys (r = 0.21 and 0.24, respectively). However, the best relation between AD-SoS and BMI was obtained by the cubic model (r^2^ = 0.13 for girls and r^2^ = 0.10 for boys) instead of linear (r^2^ = 0.04 and r^2^ 0.06, for girls and boys, respectively). Considering the results observed in AD-SoS centiles with weight, we subjectively decided to divide the sample of boys and girls in two groups according to body weight. The cut-off for the groups was set at 65 kg (< 65 kg and ≥ 65 kg) for girls and 75 kg (< 75 kg and ≥ 75 kg) for the boys, because these values are the boundaries between the categories 60–70 kg and 70–80 kg for girls and boys, respectively. Correlations between AD-SoS (m/s and Z-score) and age and anthropometric variables for total sample divided by sex and weight categories, <65 kg and ≥65 kg for girls and <75 kg and ≥75 kg for boys, are presented in detail in the S4 Table.

**Fig 4:**
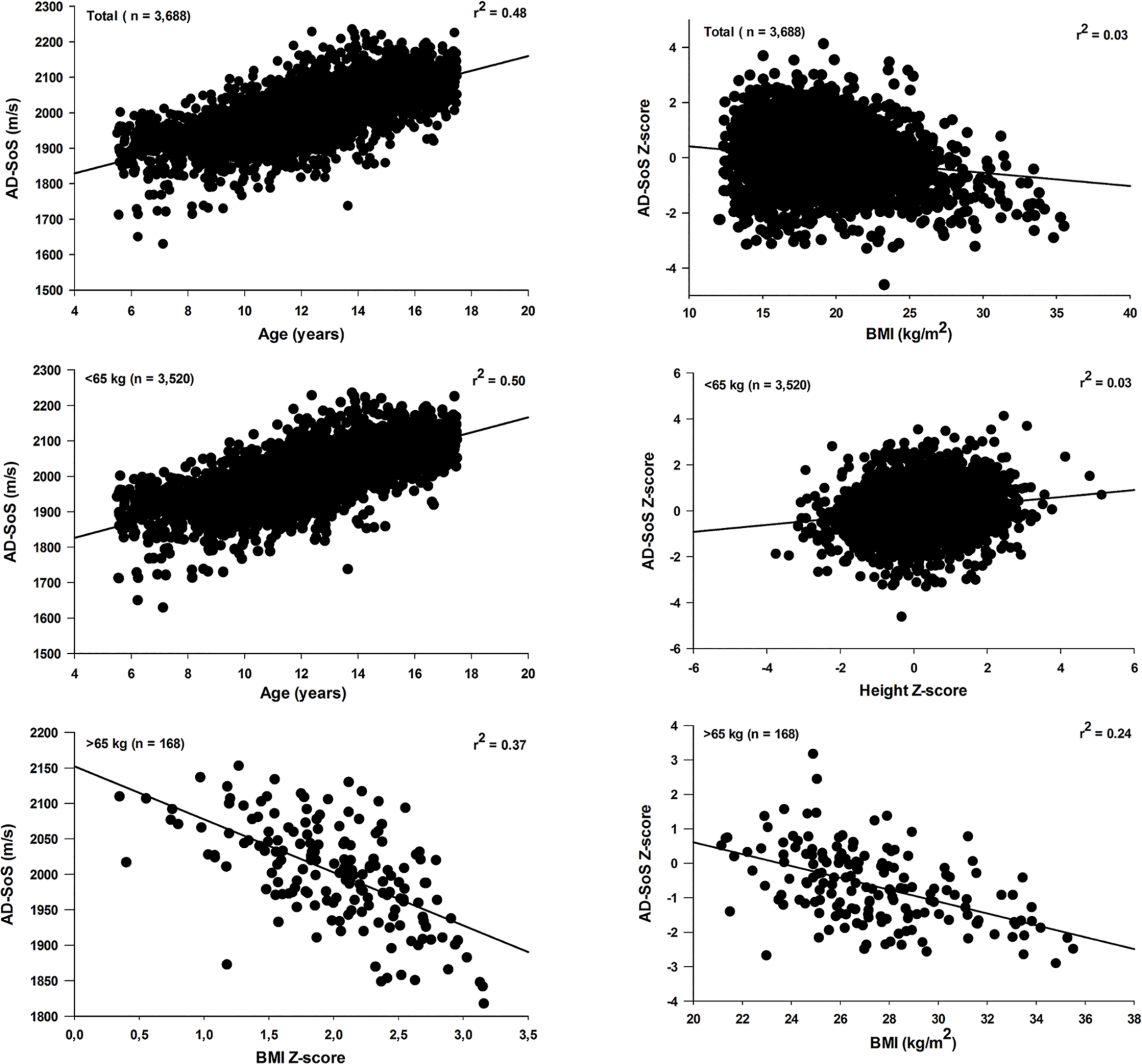
Relationship of AD-SoS (left panel) and AD-SoS Z-score (right panel) versus independents variables for total sample and divided by weight categories in girls.

The results of multiple linear regression analysis for total sample and after division by weight categories are illustrated in Figs 4 (Girls) and 5 (Boys). For girls, age accounted for 48% (beta = 0.692; p < 0.001) and 50% (beta = 0.707; p < 0.001) of AD-SoS (m/s) variance, in whole-group and those with <65 kg, respectively. For boys, height was the best predictor of AD-SoS in whole-group (beta = 0.593; p < 0.001) and among boys with less than 75 kg (beta = 0.585; p < 0.001).

**Fig 5:**
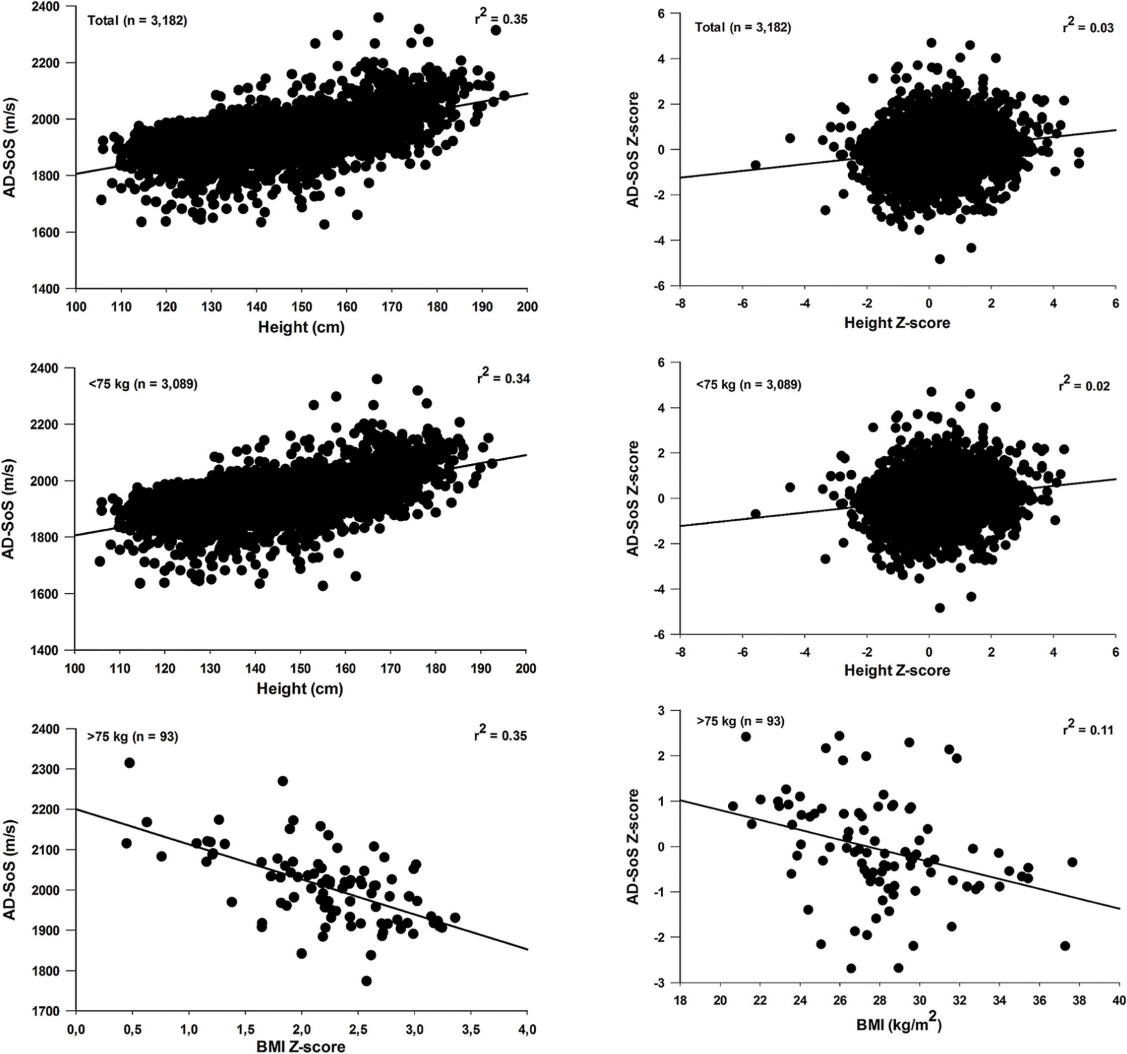
Relationship of AD-SoS (left panel) and AD-SoS Z-score (right panel) versus independents variables for total sample and divided by weight categories in boys.

When analyzing the groups of individuals with lower body weight (< 65 kg and < 75 kg, for girls and boys, respectively) and total samples, in both sexes the independent variables tested showed low predictive power for the AD-SoS Z-score, with the value of r^2^ ranging from 0.02 to 0.03. However, among heavier girls and boys (≥ 65 kg and ≥ 75 kg, respectively) similar results were observed: BMI Z-score and BMI (kg/m^2^) showed a significant negative correlation with AD-SoS (m/s) and AD-SoS Z-score, respectively, and independent predictors of AD-SoS (BMI Z-score: beta = -0.608; p < 0.001 for girls and -0.596; p < 0.001 for boys) and AD-SoS Z-score (BMI: beta = -0.499; p < 0.001 and -0.340; p < 0.001, for girls and boys, respectively).

## Discussion

Several studies demonstrate that AD-SoS parameter in healthy children and adolescents increase with age [14–18,20,25,36]. In the present study, increases in AD-SoS from 6 to 17 years were approximately 10% in both sexes. Girls presented higher values of Ad-SoS in all age groups compared to boys, except for the age group of 6 and 17 years. These findings are probably due to the later manifestation of puberty in boys. These results are consistent with other studies that verify puberty and gender influence in AD-SoS [15,18]. Puberty changes body composition and at this stage sexual dimorphism becomes clear. Bone mass acquisition reaches its peak on puberty, earlier in females than males [1], influenced by sexual hormones, GH-IGF1 axis, insulin and leptin [37].

Besides considering weight, age and gender influences, bone mineral content also varies depend on race or skin color [23]. In Brazil, miscegenation is an important aspect and must always be considered in racial analyses. Since 1991 the country has officially adopted the proposal that these data should be collected based on self-declaration; i.e., each individual chooses from five race categories—white, black, mulatto, yellow, and indigenous—which he or she feels is appropriate [29]. Brazilians are one of the most heterogeneous populations in the world, as the result of more than five centuries of miscegenation of people from different ethnic groups from several continents (Europe, Africa and Asia). In addition, around 2.5 million American Indians were already in the country. Probably, due to this great miscegenation, we did not find the influence of skin color on bone mass in this study.

Regarding the weight status, this study showed lower values of AD-SoS Z-scores in obese when compared to normal and thin boys and girls. These results are consistent with other studies using QUS of phalanges, in which obese children and adolescents showed low bone quality and reduced strength compared to controls [25], and a negative correlation between body fat and AD-SoS was found in healthy children and adolescents [24]. In our study, impairment in AD-SoS Z-scores was observed mainly among obese girls, and it was verified that in heavier children and adolescents BMI influenced negatively AD-SoS values (Figs 4 and 5 and S4 Table). The reason of the influence of obesity on bone status in children and adolescents is still unclear. Previous studies proposed a protective effect of overweight on low bone mass based on the observation of decreased risk for fragility fractures and increased bone mineral density in obese adults. The same hypothesis was also considered for pediatric age-groups.

Obese children and adolescents may have several factors impairing bone mass. Results from DXA in obese adolescents demonstrated that, despite increased mechanical loading and independent of lean mass, adiposity is not beneficial to bone structure [38]. Recently, a negative effect of fat mass was observed in bone mass development in male adolescents, and in femur and spine bone mineral density among females [26].

However, several studies have demonstrated effect of soft tissue thickness on AD-SoS measurement [39–42]. The impairment in the speed of the ultrasound wave can be misinterpreted as a reduction in the quality of the bone, while it is likely just an artifact from increased subcutaneous fat [43]. In our study it is clear that obesity negatively influences AD-SoS values in children and adolescents, but to confirm this it would be necessary to have another method (i.e., DXA) or parameter independent of the thickness of soft tissue, such as bone transmission time (BTT), unlike AD-SoS, BTT is largely independent of ultrasound attenuation and soft tissue bias [44]. In our study, we are not able to establish if this occurred due to the higher attenuation of ultrasound wave velocity in these individuals or by the negative impact of obesity in bone mass. We decided to remove the BTT, in our study, because we lost about 30% of the data of this variable and we considered that this could interfere with the comparisons with AD-SoS results.

The new database provided in this study of QUS parameters of the phalanges can be a useful tool to assess the position of an individual, compared to a reference population and analyzing the trajectory of these parameters in longitudinal assessments [11]. However, it’s important to highlight that we cannot claim that individuals with lower values of AD-SoS for age, height or weight (ie: <- 2 or z-score <10th centile) may have increased risk of bone fracture than individuals considered "normal" (AD-SoS Z-scores values greater than 0). Our study did not evaluate the risk of fracture, until now the only validated skeletal site for the clinical use of QUS in the management of osteoporosis is the heel [45]. A recent prospective study demonstrated that AD-SoS is significantly independent predictor of osteoporotic fractures (hip and clinical vertebral fractures) in postmenopausal women and it was able to discriminate between fractured and non-fractured subjects in the group of women [46]. Moreover, previous studies indicated that QUS might be a useful method to indentify bone fracture risk in pediatric patients with bone and mineral disorders. [4,47]. Considering this findings, our data could be used as an initial screen for low bone mass among children and adolescents.

Our study has some limitations, such as the lack of a reference method, such as DXA, to determine more accurately if the negative influence found by the analyzed ultrasound parameter (AD-SoS) reflects negative effect of obesity on bone mass or a limitation of this parameter in assessing these individuals. Additionally, there is lack of information on past bone fractures, sex hormones measurements and physical activity level, which could enhance the results. Nevertheless, the absence of such measures can be justified in part by the difficulties to obtain those informations in a large number of individuals, particularly in children and adolescents.

However, this study provides a large number of subjects investigated and the use of a relatively simple and portable tool for assessment of bone status. This can facilitate the reproducibility of our findings in groups of individuals with similar characteristics as schools around the world. Moreover, QUS assesses not only quantity but also the quality of bone based on the information of the micro-architecture and elasticity [9,10,12]. The relative simplicity, ease of transportation and non-exposure to radiation, presents advantages for the use of QUS in comparison to other methods in children and adolescents [5].

## Conclusions

In our sample of girls and boys aged 7–17 years, age and height, respectively were main determinants of AD-SoS. The present study reports reference AD-SoS values in healthy children and adolescents based on a representative and the largest sample ever published. This parameter proved to be sensitive to changes influenced by sex, age and weight status, but not by skin color. These results indicate a negative influence of obesity on AD-SoS. In this sense, we recommend that the results of AD-SoS in obese children and adolescents should be interpreted with caution. However, our normative data could be used for monitoring bone status in individual or samples with age range from seven to 17 years.

## Supporting Information

S1 Table LMS coefficients and smoothed percentiles (3th, 10th, 25th, 75th, 90th and 97th) of AD-SoS (m/s) for Brazilian children and adolescents according to age (years) and sex. (DOCX)

S2 Table LMS coefficients and smoothed percentiles (3th, 10th, 25th, 75th, 90th and 97th) of AD-SoS (m/s) for Brazilian children and adolescents according to height (cm) and sex. (DOCX)

S3 Table LMS coefficients and smoothed percentiles (3th, 10th, 25th, 75th, 90th and 97th) of AD-SoS (m/s) for Brazilian children and adolescents according to weight (kg) and sex. (DOCX)

S4 Table Correlation coefficients between AD-SoS and age and anthropometrics measurements for Brazilian children and adolescents according to weight categories and sex. (DOCX)
