# Supplementary material for: Brazilian Pediatric Reference Data for Quantitative Ultrasound of Phalanges According to Gender, Age, Height and Weight
Source: PLoS One. 2015 Jun 4;10(6):e0127294. doi: 10.1371/journal.pone.0127294 (PMC4456168; doi:10.1371/journal.pone.0127294)
Supplement: S1 Table — (DOCX) [file pone.0127294.s001.docx]

**Supplemental Data**

**Suppl. Table 1:** LMS coefficients and smoothed percentiles (3th, 10th, 25th, 75th, 90th and 97th) of AD-SoS (m/s) for Brazilian children and adolescents according to age (years) and sex.

|  | Sample |  |  |  | Smoothed percentile | | | | | |
| --- | --- | --- | --- | --- | --- | --- | --- | --- | --- | --- |
| Age | Size | *L* | *M (*50th) | *S* | 3th | 10th | 25th | 75th | 90th | 97th |
| *Girls* |  |  |  |  |  |  |  |  |  |  |
| 6 | 84 | 10,596 | 1901 | 0,028 | 1747 | 1813 | 1862 | 1934 | 1962 | 1987 |
| 7 | 150 | 9,330 | 1909 | 0,028 | 1767 | 1825 | 1871 | 1942 | 1971 | 1996 |
| 8 | 167 | 7,552 | 1922 | 0,027 | 1793 | 1842 | 1885 | 1954 | 1984 | 2011 |
| 9 | 334 | 5,497 | 1937 | 0,027 | 1818 | 1861 | 1901 | 1970 | 2001 | 2030 |
| 10 | 421 | 3,605 | 1952 | 0,027 | 1839 | 1878 | 1916 | 1986 | 2019 | 2050 |
| 11 | 489 | 2,124 | 1964 | 0,027 | 1853 | 1891 | 1928 | 2000 | 2035 | 2069 |
| 12 | 515 | 0,805 | 1985 | 0,029 | 1870 | 1908 | 1947 | 2024 | 2063 | 2103 |
| 13 | 487 | 0,864 | 2017 | 0,031 | 1893 | 1934 | 1976 | 2059 | 2101 | 2143 |
| 14 | 370 | 1,903 | 2046 | 0,030 | 1918 | 1961 | 2004 | 2087 | 2127 | 2167 |
| 15 | 310 | 2,866 | 2066 | 0,028 | 1943 | 1985 | 2026 | 2103 | 2140 | 2176 |
| 16 | 226 | 3,655 | 2083 | 0,026 | 1967 | 2008 | 2046 | 2118 | 2151 | 2183 |
| 17 | 135 | 4,165 | 2092 | 0,024 | 1982 | 2021 | 2057 | 2125 | 2157 | 2187 |
| *Boys* |  |  |  |  |  |  |  |  |  |  |
| 6 | 103 | 10,698 | 1888 | 0,028 | 1733 | 1800 | 1849 | 1920 | 1948 | 1972 |
| 7 | 239 | 10,549 | 1889 | 0,028 | 1735 | 1801 | 1850 | 1921 | 1949 | 1974 |
| 8 | 331 | 9,807 | 1894 | 0,029 | 1742 | 1806 | 1854 | 1927 | 1956 | 1982 |
| 9 | 308 | 8,271 | 1905 | 0,029 | 1757 | 1816 | 1864 | 1939 | 1970 | 1998 |
| 10 | 388 | 6,641 | 1915 | 0,030 | 1772 | 1827 | 1874 | 1952 | 1985 | 2016 |
| 11 | 386 | 5,038 | 1925 | 0,031 | 1785 | 1837 | 1883 | 1964 | 2000 | 2033 |
| 12 | 350 | 3,202 | 1937 | 0,033 | 1800 | 1848 | 1894 | 1978 | 2018 | 2056 |
| 13 | 361 | 1,080 | 1955 | 0,034 | 1820 | 1865 | 1910 | 1999 | 2044 | 2089 |
| 14 | 307 | -0,977 | 1983 | 0,036 | 1850 | 1892 | 1937 | 2032 | 2083 | 2137 |
| 15 | 203 | -2,345 | 2013 | 0,037 | 1881 | 1922 | 1966 | 2065 | 2121 | 2182 |
| 16 | 136 | -3,274 | 2045 | 0,036 | 1917 | 1956 | 1998 | 2098 | 2156 | 2222 |
| 17 | 70 | -4,285 | 2085 | 0,035 | 1961 | 1998 | 2039 | 2137 | 2196 | 2265 |

*L*, Box-Cox transformation power; *M*, median; *S*, generalized coefficient of variation.
