# Supplementary material for: Brazilian Pediatric Reference Data for Quantitative Ultrasound of Phalanges According to Gender, Age, Height and Weight
Source: PLoS One. 2015 Jun 4;10(6):e0127294. doi: 10.1371/journal.pone.0127294 (PMC4456168; doi:10.1371/journal.pone.0127294)
Supplement: S2 Table — (DOCX) [file pone.0127294.s002.docx]

**Supplemental Data**

**Suppl. Table 2:** LMS coefficients and smoothed percentiles (3th, 10th, 25th, 75th, 90th and 97th) of AD-SoS (m/s) for Brazilian children and adolescents according to height (cm) and sex.

|  | Sample |  |  |  | Smoothed percentile | | | | | |
| --- | --- | --- | --- | --- | --- | --- | --- | --- | --- | --- |
| Height | Size | *L* | *M (50th)* | *S* | 3th | 10th | 25th | 75th | 90th | 97th |
| *Girls* |  |  |  |  |  |  |  |  |  |  |
| 110 | 52 | 11.900 | 1891 | 0.027 | 1739 | 1806 | 1854 | 1922 | 1948 | 1971 |
| 120 | 220 | 8.746 | 1912 | 0.027 | 1777 | 1831 | 1875 | 1945 | 1973 | 1999 |
| 130 | 473 | 5.504 | 1934 | 0.028 | 1809 | 1855 | 1897 | 1969 | 2001 | 2031 |
| 140 | 662 | 2.575 | 1954 | 0.029 | 1835 | 1876 | 1916 | 1992 | 2028 | 2063 |
| 150 | 855 | 0.258 | 1992 | 0.031 | 1870 | 1910 | 1951 | 2034 | 2077 | 2120 |
| 160 | 1081 | 1.569 | 2040 | 0.032 | 1907 | 1952 | 1997 | 2084 | 2127 | 2169 |
| 170 | 330 | 3.090 | 2066 | 0.031 | 1928 | 1976 | 2022 | 2108 | 2148 | 2187 |
| 180 | 15 | 4.349 | 2085 | 0.030 | 1945 | 1995 | 2042 | 2125 | 2163 | 2199 |
| *Boys* |  |  |  |  |  |  |  |  |  |  |
| 110 | 52 | 10.259 | 1876 | 0.029 | 1717 | 1786 | 1836 | 1910 | 1939 | 1964 |
| 120 | 323 | 8.726 | 1887 | 0.030 | 1735 | 1797 | 1847 | 1922 | 1953 | 1980 |
| 130 | 568 | 6.830 | 1901 | 0.031 | 1756 | 1812 | 1860 | 1938 | 1971 | 2001 |
| 140 | 679 | 4.622 | 1917 | 0.032 | 1779 | 1829 | 1875 | 1956 | 1993 | 2027 |
| 150 | 577 | 2.545 | 1933 | 0.033 | 1799 | 1846 | 1890 | 1975 | 2016 | 2055 |
| 160 | 432 | 0.169 | 1961 | 0.035 | 1828 | 1871 | 1916 | 2007 | 2054 | 2102 |
| 170 | 384 | -1.376 | 2014 | 0.037 | 1878 | 1921 | 1966 | 2065 | 2119 | 2176 |
| 180 | 145 | -1.482 | 2054 | 0.037 | 1914 | 1958 | 2005 | 2107 | 2164 | 2224 |
| 190 | 22 | -1.425 | 2094 | 0.038 | 1949 | 1995 | 2043 | 2149 | 2207 | 2269 |

*L*, Box-Cox transformation power; *M*, median; *S*, generalized coefficient of variation.
