# Supplementary material for: Brazilian Pediatric Reference Data for Quantitative Ultrasound of Phalanges According to Gender, Age, Height and Weight
Source: PLoS One. 2015 Jun 4;10(6):e0127294. doi: 10.1371/journal.pone.0127294 (PMC4456168; doi:10.1371/journal.pone.0127294)
Supplement: S3 Table — (DOCX) [file pone.0127294.s003.docx]

**Supplemental Data**

**Suppl. Table 3:** LMS coefficients and smoothed percentiles (3th, 10th, 25th, 75th, 90th and 97th) of AD-SoS (m/s) for Brazilian children and adolescents according to weight (kg) and sex.

|  | Sample |  |  |  | Smoothed percentile | | | | | |
| --- | --- | --- | --- | --- | --- | --- | --- | --- | --- | --- |
| Weight | Size | *L* | *M (50th)* | *S* | 3th | 10th | 25th | 75th | 90th | 97th |
| *Girls* |  |  |  |  |  |  |  |  |  |  |
| 20 | 350 | 7.104 | 1920 | 0.030 | 1776 | 1832 | 1879 | 1956 | 1989 | 2018 |
| 30 | 927 | 3.506 | 1951 | 0.029 | 1829 | 1872 | 1912 | 1987 | 2023 | 2056 |
| 40 | 893 | 0.896 | 1992 | 0.034 | 1856 | 1902 | 1947 | 2037 | 2083 | 2128 |
| 50 | 917 | 3.138 | 2042 | 0.035 | 1887 | 1942 | 1993 | 2089 | 2134 | 2176 |
| 60 | 433 | 3.400 | 2042 | 0.035 | 1886 | 1941 | 1993 | 2088 | 2132 | 2174 |
| 70 | 124 | 4.793 | 2021 | 0.034 | 1864 | 1922 | 1974 | 2065 | 2105 | 2143 |
| 80 | 35 | 6.475 | 1987 | 0.031 | 1838 | 1895 | 1944 | 2026 | 2061 | 2092 |
| 90 | 9 | 8.619 | 1930 | 0.024 | 1816 | 1860 | 1898 | 1959 | 1985 | 2009 |
| *Boys* |  |  |  |  |  |  |  |  |  |  |
| 20 | 415 | 9.491 | 1888 | 0.030 | 1725 | 1794 | 1846 | 1923 | 1954 | 1981 |
| 30 | 978 | 5.120 | 1913 | 0.032 | 1772 | 1824 | 1870 | 1951 | 1987 | 2020 |
| 40 | 693 | 1.981 | 1933 | 0.033 | 1801 | 1846 | 1890 | 1976 | 2017 | 2058 |
| 50 | 488 | -0.037 | 1958 | 0.037 | 1817 | 1862 | 1909 | 2007 | 2058 | 2110 |
| 60 | 346 | 0.440 | 1998 | 0.050 | 1819 | 1878 | 1937 | 2060 | 2122 | 2186 |
| 70 | 169 | 0.888 | 2022 | 0.047 | 1833 | 1896 | 1959 | 2085 | 2149 | 2212 |
| 80 | 64 | 0.955 | 2019 | 0.047 | 1832 | 1894 | 1957 | 2082 | 2145 | 2207 |
| 90 | 20 | 1.112 | 2000 | 0.046 | 1821 | 1881 | 1941 | 2061 | 2120 | 2179 |
| 100 | 9 | 1.214 | 1988 | 0.043 | 1815 | 1873 | 1931 | 2045 | 2101 | 2158 |

*L*, Box-Cox transformation power; *M*, median; *S*, generalized coefficient of variation.
