# Supplementary material for: Brazilian Pediatric Reference Data for Quantitative Ultrasound of Phalanges According to Gender, Age, Height and Weight
Source: PLoS One. 2015 Jun 4;10(6):e0127294. doi: 10.1371/journal.pone.0127294 (PMC4456168; doi:10.1371/journal.pone.0127294)
Supplement: S4 Table — (DOCX) [file pone.0127294.s004.docx]

**Supplemental Data**

**Suppl. Table 4:** Correlation coefficients between AD-SoS and age and anthropometrics measurements for Brazilian children and adolescents according to weight categories and sex.

|  |  | |  | GIRLS | |  |  | |  |  | |  | BOYS | |  |  | |
| --- | --- | --- | --- | --- | --- | --- | --- | --- | --- | --- | --- | --- | --- | --- | --- | --- | --- |
|  | Total (n=3,688) | |  | <65 kg (n=3,520) | |  | ≥65 kg (n=168) | |  | Total (n=3,182) | |  | <75 kg (n=3,089) | |  | ≥75 kg (n=93) | |
|  | AD-SoS | |  | AD-SoS | |  | AD-SoS | |  | AD-SoS | |  | AD-SoS | |  | AD-SoS | |
|  | *m/s* | *Z-score* |  | *m/s* | *Z-score* |  | *m/s* | *Z-score* |  | *m/s* | *Z-score* |  | *m/s* | *Z-score* |  | *m/s* | *Z-score* |
| Age (yrs) | 0.69^a^ | 0.01 |  | 0.71^a^ | 0.04^a^ |  | 0.40^a^ | -0.18 |  | 0.59^a^ | 0.004 |  | 0.58^a^ | 0.01 |  | 0.48^a^ | -0.06 |
| Weight (kg) | 0.48^a^ | -0.06 ^a^ |  | 0.56^a^ | 0.03 |  | -0.35^a^ | -0.42^a^ |  | 0.50^a^ | 0.02 |  | 0.51^a^ | 0.03 |  | -0.13 | -0.21^b^ |
| Height (cm) | 0.64^a^ | 0.08 ^a^ |  | 0.66^a^ | 0.11^a^ |  | 0.43^a^ | 0.26^a^ |  | 0.59^a^ | 0.07^a^ |  | 0.59^a^ | 0.08^a^ |  | 0.52^a^ | 0.26^b^ |
| BMI (kg/m2) | 0.21^a^ | -0.17^a^ |  | 0.27^a^ | -0.09^a^ |  | -0.56^a^ | -0.50^a^ |  | 0.24^a^ | -0.06^a^ |  | 0.22^a^ | -0.05^b^ |  | -0.49^a^ | -0.34^a^ |
| Height Z-score | 0.01 | 0.15^a^ |  | 0.001 | 0.14^a^ |  | 0.14 | 0.36^a^ |  | 0.07^a^ | 0.16^a^ |  | 0.06^b^ | 0.16^a^ |  | 0.02 | 0.30^a^ |
| BMI Z-score | -0.10^a^ | -0.15^a^ |  | -0.11^a^ | -0.10^a^ |  | -0.61^a^ | -0.38^a^ |  | -0.001 | -0.04^b^ |  | -0.04^b^ | -0.03 |  | -0.60^a^ | -0.31^a^ |

BMI, body mass index.

^a^ p< 0.01. ^b^ p< 0.05.
